# Perceived wellbeing of patients one year post stroke in general practice - *recommendations for quality aftercare*

**DOI:** 10.1186/1471-2377-11-42

**Published:** 2011-03-31

**Authors:** Leonie de Weerd, Wijnand AF Rutgers, Klaas H Groenier, Klaas van der Meer

**Affiliations:** 1 Department of General Practice, University Medical Center Groningen, Antonius Deusinglaan 1, 9713 AV, Groningen, the Netherlands; 2 Department of Neurology, Martini Hospital Groningen, Van Swietenplein 1, 9700 RM, Groningen, the Netherlands

## Abstract

**Background:**

Annually, 41,000 people in the Netherlands have strokes. This has multiple physical and psychosocial consequences. Most patients return home after discharge from hospital. Quality aftercare by general practitioners is important to support patients at home. The purpose of this study is to examine the wellbeing of patients who returned home immediately after discharge from hospital, one year post stroke, in comparison with the general Dutch population of the same age and to determine factors that could influence wellbeing.

**Methods:**

All the stroke patients from the Department of Neurology, Martini Hospital Groningen in the period November 2006 to October 2007 were included. People aged under 65 years or with haemorrhaging were excluded. All the patients (N = 57) were interviewed at home using the following questionnaires: Barthel Index, SF-36, HADS, CSI and a questionnaire about their way of life.

**Results:**

31% of the patients in this study experienced a decrease in functional status after one year. Nevertheless, there was no significant difference between the median Barthel Index value at discharge from hospital and one year post stroke. ADL independence correlated with a better quality of life. The health-related quality of life was high. Stroke patients have almost the same quality of life as the 'average' Dutch elderly population. Where patients can no longer fully participate in society, their perceived quality of life is also lower. In this study there is an indication of a high prevalence of depression and anxiety disorders in stroke patients. This negatively affects the quality of life a year after stroke. Although caregiver strain was low for the partners of stroke patients, a reduced quality of life is correlated to greater burden.

**Conclusions:**

This study provides valuable insight into the wellbeing of patients living at home one year post stroke. Physical functioning and quality of life are comparable to the general population of the same age, but improvements in mental functioning can be envisaged. In addition, more attention should be paid to maintaining the patients' activities. The wellbeing of these stroke patients could be increased further if greater attention is paid to these aspects of life. This seems to be applicable to general practice.

## Background

About 41,000 people in the Netherlands are affected by strokes each year [1,2]. Men and women have similar absolute lifetime stroke risks [3]. 25% of all patients die in the first year after a stroke [4]. Mean age at onset is 70 years in men and 75 years in women [5].

Strokes affect patients' lives in many different ways, not only physically but also through a range of emotional, psychic, cognitive and social consequences. The seriousness of post-stroke physical and mental impairments influences quality of life. As stroke mortality declines, more patients have to live with multiple handicaps and impairments. Therefore, improving the quality of life and paying greater attention to rehabilitation is increasingly important [6-8].

The post-stroke period can be divided into three phases, namely the acute phase, the rehabilitation phase and the chronic phase. It is important that these phases be distinguished because they differ in the treatment and support of patients. This study concerns the chronic post-stroke phase, which starts approximately half a year after a stroke. This phase involves acceptance and coping with persistent disabilities [9].

Most patients return home after discharge from hospital. After discharge, patients in the Netherlands must visit their general practitioner (GP) when they experience physical and psychical discomfort. GPs can assist patients with coping with disabilities and psychic problems, even long after the stroke. Furthermore, GPs handle secondary prevention [10]. Accordingly, it is important for GPs to know the primary care outcomes and what makes a good prognosis long after a stroke [7,8]. Only in this way can GPs assist with improving physical and psychical functioning and quality of life.

Little is known about the outcomes for Dutch patients for longer periods post stroke. Most studies are of outcomes in the acute or rehabilitation phase [11]. Therefore, it is important to determine the wellbeing of patients in the chronic phase and to define which factors can improve their quality of life.

As little is known about late physical and psychical consequences of ischemic stroke in primary care patients in the Netherlands, it is hard to decide whether patients in the chronic phase receive quality aftercare. It is logical that physical and psychical stroke outcomes should be related to quality of life. Therefore, to improve quality of life, knowledge of outcomes is important. Only then can future GP interventions be focused on improving quality of life. The purpose of this study is to examine the wellbeing of patients one year post stroke who returned to their home immediately after discharge from hospital and to determine factors that can influence wellbeing. Health-related quality of life is compared to the health-related quality of life of the general Dutch population of the same age.

## Methods

### Study design

The study includes all the ischemic stroke patients admitted to the Department of Neurology, Martini Hospital Groningen, between November 2006 and October 2007. A total of 244 patients were included. The exclusion criteria for patients were being under 65 years or moving on to a nursing home, rehabilitation centre or other hospital department after being discharged from hospital. People already living in a retirement home before their stroke were included in the study. The MEC (medical ethical committees) of the Martini Hospital has approved this study (17-01-2008). Informed consent was obtained from all patients.

After informed consent, clinical details including stroke severity, history, comorbidity, risk factors (present before stroke), use of medication and demographic information were obtained from the medical records. Stroke severity was determined by the National Institute of Health Stroke Scale (NIHSS) examination. The comorbidity, history and risk factors recorded were hypertension, prior myocardial infarction, prior transient ischemic attack (TIA) or strokes, diabetes, hypercholesterolemia, atrial fibrillation, cognitive impairment, prior depression or anxiety disorder and smoking. The demographic information collected was age, gender and social status.

One year post stroke (January 2008 to October 2008) all the patients were visited at home by one medical practitioner. Patients were interviewed in person by a trained medical practitioner and standardized questionnaires were administered.

### Measures at 12 months

The Barthel Index (BI) was used to assess disability in our patients. The BI measures independence in daily living activities and yields a score ranging from 0 (functionally dependent) to 20-21 (functionally independent). In this questionnaire patients are given three points for eating independently, rather than two points. The sensitivity and reliability of the BI are high for stroke patients [12-14].

To measure health-related quality of life (HRQOL) we used the Short Form 36 (SF-36). The SF-36 consists of 36 questions and comprises 8 health scales (physical function [FF], role limitations - physical [Rlf], social functioning [SF], role limitations - emotional [Rle], bodily pain [BP], general health [GH], vitality [Vit] and mental health [MH]). The original 0-100 scoring algorithm (ranging from 0 [poor HRQOL] to 100 [good HRQOL]) was used based on the summated ratings method. The SF-36 is a reliable and valid measure for determining HRQOL in stroke patients [15-20]. The HRQOL in this study was compared to the HRQOL of the Dutch elderly population [16].

To identify the possible and probable presence of depression and anxiety disorders in our study the Hospital Anxiety and Depression Scale (HADS) was used. The HADS is a 14-item scale divided into depression and anxiety subscales. The possible scores for depression or anxiety range from 0 to 21. A score of 8 to 10 means possible anxiety disorder or depression and a score of 11 or higher indicates the probable presence of a mood disorder [21,22].

Caregiver strain was assessed with the Caregiver Strain Index (CSI). This 13-item questionnaire is a valid instrument for determining the burden on the spouses of stroke patients [23,24].

In addition, patients were asked (multiple choice) if they had changed their activities in relation to the following habits and daily occupations: 1) smoking frequency (unchanged, ceased, less or more), 2) alcohol consumption (unchanged, ceased, less or more), 3) housekeeping (unchanged, ceased, less, more), 4) physical exercise (unchanged, ceased, less or more), 5) hobbies (unchanged, ceased, less or more), 6) reading (unchanged, ceased, less or more), 7) visiting family and friends (unchanged, ceased, less or more), 8) membership of clubs or associations (unchanged, ceased or joined new) and 9) going on holidays (unchanged, ceased, less or more).

### Statistical analysis

SPSS 15 for Windows was used for statistical analysis. Statistical significance was set at p < 0.05 (2-sided). For comparisons between groups we used the following parametric and non-parametric tests: Student's *t*-test, one-way ANOVA, Mann-Whitney U test, Kruskall-Wallis, and Chi-square tests for independent observations. Student's paired t-test and Wilcoxon signed rank test were used for correlated observations.

Since the number of observations is rather low, deviations of normality of the Barthel Index could not be assessed in a proper way. Therefore we used non-parametric tests for testing differences of the Barthel Index.

To describe the number of patients who changed their habits due to stroke the responses were categorized into 'more', 'less', 'as much as before' or 'quit'. Pearson's and Spearman's rank correlations were used to measure the impact of different variables on functioning (BI) and health-related quality of life.

Despite the relatively large number of statistical tests applied, we decided to do no correction for 'multiple testing' (for instance by means of the Bonferroni method). Instead, the p-values are merely given as an indication of the strength of the evidence.

## Results

**Figure 1:**
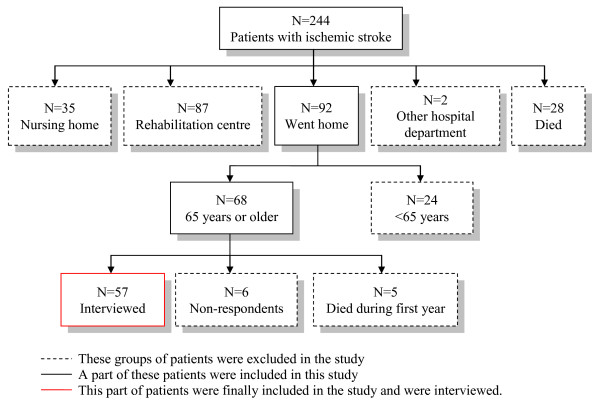
**Flow chart of selection of patients**.

244 patients were found to have had an ischemic stroke during the study period. 92 patients went home immediately after discharge. 68 of these patients were 65 years or older. The response rate was 57 (84%)(see Figure 1). We could not complete the HADS questionnaire for two patients and the daily occupations questionnaire for one because these patients did not know how to the answer the questions.

The mean age of the study population was 77 years and 44% of the patients were men. Most patients had had a stroke on the left side of the brain (61%). Hypertension and smoking were most often present as risk factors. A quarter of the patients have a history of having a TIA/stroke and a quarter had ischemic heart disease before their stroke. The detailed baseline characteristics of the study population are provided in Table 1.

**Table 1:** Baseline characteristics of the study population

Variables	Number (%)
Patients included	57
Gender	
- *Men*	25 (44%)
- *Women*	32 (56%)
Age, average (range)	77 (65-91)
Living situation	
- *Living alone*	29 (51%)
- *Living with a partner*	28 (49%)
Type of stroke	
- *Lacunar Circulation Infarct*	24 (42%)
- *Partial Anterior Circulation Infarct*	21 (37%)
- *Posterior Circulation Infarct*	8 (14%)
- *Total Anterior Circulation Infarct*	0 (0%)
- *Unknown*	4 (7%)
Brain hemisphere	
- *Left side*	35 (61%)
- *Right side*	17 (30%)
- *Unknown*	5 (9%)
NIHSS, average (range)	3 (0-20)
Barthel Index, median (range)	
- *At discharge*	21 (10-21)
- *After 1 year*	21 (13-21)
Risk factors present before stroke	
- *No risk factors*	11 (19%)
- *Hypertension*	29 (51%)
- *Hypercholesterolemia*	11 (19%)
- *Diabetes*	12 (21%)
- *Cardiac embolus*	10 (18%)
- *Smoking*	16 (28%)
History	
- *No history*	6 (11%)
- *Ischemic heart disease*	14 (25%)
- *Stroke/TIA*	14 (25%)
- *Atrial fibrillation*	5 (9%)
- *Heart failure*	3 (5%)
- *COPD*	10 (18%)
- *Depression*	2 (4%)
- *Mild cognitive impairment*	3 (5%)
- *Abuse of alcohol*	3 (5%)

### Functioning

None of the patients who went home immediately after discharge were totally or severely dependent. Most patients at discharge (98.2%) and one year after stroke (96.5%) were not dependent at all or only slightly dependent. 50% of the patients scored the same level of dependency at discharge and one year post stroke. 14% had better scores one year post stroke and 32% had worse scores. Following statistical analyses, there is no significant difference in the median BI score at discharge and one year post stroke (p = 0.304). There is no significant difference between men and women. Patients in retirement homes score worse on the BI than patients living alone (Table 2).

**Table 2:** Functioning after one year according to demographic factors, impairment, disability and change in activities

Variables		N	Median Barthel Index	Range	P
* Barthel Index*^*1*^	At discharge	57	21	10-21	0,304
	After 1 year	57	21	13-21	
					
*Gender*^*3*^	Male	25	21	13-21	0,098
	Female	32	20	14-21	
					
* Social situation*^*2*^	Living alone	28	21	16-21	**0,044**
	Living with partner	27	21	13-21	
	Home for elderly	2	15,5	14-17	
					
* Housekeeping*^*2*^	As much as before	25	21	14-21	**<0,001**
	Quit	9	18	13-21	
	Less	17	19	15-21	
	More	5	21	19-21	
					
*Physical activity*^*2*^	As much as before	18	21	17-21	**0,003**
	Quit	4	15	13-18	
	Less	32	21	15-21	
	More	2	21	21-21	
					
*Hobbies*^*2*^	As much as before	35	21	14-21	0,660
	Quit	1	19	19-19	
	Less	19	21	13-21	
	More	1	21	21-21	
					
*Visiting*^*2*^	As much as before	38	21	16-21	**0,001**
	Quit	3	15	13-18	
	Less	15	20	14-21	
	More	0	.		
					
*Experienced quality of life*^*3*^	The same	38	21	14-21	0,560
	Diminished	18	20,5	13-21	
					
*Depression*^*2#*^	None	48	21	14-21	0,057
	Possible	4	18,5	13-20	
	Probable	3	20	18-21	
					
*Anxiety*^*2#*^	None	50	21	13-21	0,489
	Possible	2	19,5	18-21	
	Probable	3	20	19-20	

^1^Wilcoxon signed rank test, ^2^Kruskall-Wallis test, ^3^Mann-Whitney U test. ^# ^Based on the HADS (none: <8 points, possible: 8-10 points and probable: >10 points)

In this study, housekeeping (p < 0.001), physical activity (p = 0.003) and visiting family and friends (p = 0.001) are related to a decline in dependency. Having a depression (0,057), an anxiety disorder (p = 0.489) or an experienced decline in quality of life (p = 0.560) are not related to being more dependent (Table 2).

### Daily occupations

45% of patients could perform the same housekeeping tasks as they did before their strokes. 30% of patients could not perform the same housekeeping tasks and 16% had to stop all household activities.

Considering physical activity, 57% of patients engaged in fewer physical activities due to restrictions caused by stroke (paresis of limbs and fatigue). 7% had to cease all physical activities. Most patients tried to keep active with the aid of a physiotherapist or using a home trainer.

63% of patients had trouble maintaining their hobbies. 2% had to quit their hobbies and 34% could not do them as often as before the stroke. Hobbies such as home improvement for men and embroidery for women are were particularly difficult to maintain.

27% of patients made fewer visits to family and friends. Most often these people were living alone, could no longer use their bicycle or lost their driving licences due to their stroke. Although this percentage is high, a lot of patients (68%) reported that visiting family and friends returned to normal one year post stroke.

### Mood disorder

Of the patients in our study population 3.5% had a history of depression. There was an indication of depression one year post stroke in 12.7% of patients. About 6% of the Dutch population (aged 18-65 years) are currently depressed or have just had depression [25,26]. For the elderly the figure is 2% [27,28]. Accordingly, the patients in this study seem to suffer more often from depression than the general Dutch population. Women are affected twice as frequently as men [29]. One year post stroke there is no difference in prevalence between men and women (p = 0.590).

According to the HADS scores there is an indication for anxiety disorder in 9.1% of patients one year post stroke. Of the Dutch population (aged 18-65 years), 2.3% had experienced an anxiety disorder. Women are also twice as affected by anxiety disorder as men [26]. The twelve-month prevalence for anxiety disorder in the Dutch population is 0.7% [29,30]. This percentage increases with age. Eleven percent of people aged 65-75 years have had an anxiety disorder the preceding year [31]. This can be confirmed in this study. However, no statistical significant difference between the prevalence in men and women could be found (p = 0.383).

We compared different variables such as social status or caregiver strain with depression or anxiety disorder in this group of stroke patients. For these variables no statistical significant relationship could be found (p-values varied from 0.245 to 0.680).

### Health-related quality of life (SF-36)

Table 3 shows the comparison between the HRQOL of the patients from this study and the Dutch elderly population [16]. In this study the mean age is 77 years (range 65-91). Therefore, most patients fall into the 75-85 years category. When we compare this category to our study population, HRQOL is nearly identical, except for two health scales. Patients in this study have a significantly better HRQOL for the 'role limitations - emotional' and 'bodily pain' health scales.

**Table 3:** Comparison of the SF-36 in our study with the Dutch elderly population

SF-36	Patients in this study		**75-85 years**^**#**^		
	***Mean***	***SD***	***Mean***	***SD***	P^*^
*Physical functioning*	60,4	28,4	56,0	29,7	0,256
*Social functioning*	84,2	22,7	82,0	24,9	0,481
*Role limitations - physical*	62,1	37,5	60,1	43,1	0,698
*Role limitations - emotional*	91,1	25,8	73,7	40,4	**0,000**
*Mental Health*	76,1	17,2	76,9	14,3	0,743
*Vitality*	63,0	20,3	60,1	21,3	0,284
*Bodily Pain*	81,7	20,2	72,0	30,3	**0,001**
*General Health*	61,5	23,4	59,0	21,2	0,424

^# ^Dutch elderly population (75-85 year) *One-sample T-test

Multiple aspects of the SF-36 were related to physical functioning (Barthel Index). There is a high correlation with the health scale 'physical functioning' (R = 0.610) and a moderate correlation with 'role limitations - physical' (R = 0.412), 'mental health' (R = 0.416) and 'vitality' (R = 0.418).

Table 4 presents the relationship between HRQOL and mood or change in activities. Patients with no depression or anxiety presented a significantly higher HRQOL for the 'social functioning', 'role limitations - emotional', 'mental health' and 'vitality' health scales. Patients who stopped or reduced activities such as housekeeping and physical exercise have a significantly worse HRQOL for physical functioning and physical role limitations. Less frequent visits with family and friends is related to a diminished HRQOL (physical and social functioning, mental health and vitality) in this study.

**Table 4:** Relationship between HRQOL and mood or change in activities (statistical significant results of the independent-samples T-test or one-way ANOVA)

Variables		N	FF	p	SF	p	RLf	p	RLe	p	MH	p	Vit	p	GH	p
* Depression*^*#*^	None	48	62,0	0,284	87,5	**0,003**	64,1	0,405	96,5	**0,000**	79,7	**0,000**	66,8	**0,001**	64,0	0,062
	Possible	4	38,8		50,0		37,5		50,0		61,0		45,0		38,8	
	Probable	3	55,0		70,8		58,3		55,6		46,7		30,0		46,7	
																
* Anxiety*^*#*^	None	50	60,3	0,661	86,3	**0,009**	63,5	0,372	94,7	**0,002**	78,8	**0,000**	65,6	**0,016**	61,9	0,453
	Possible	2	42,5		81,3		25,0		50,0		82,0		45,0		67,5	
	Probable	3	65,0		45,8		58,3		55,6		34,7		35,0		45,0	
*House-keeping*	As much	25	73,4	**0,000**	92,0	**0,003**	76,0	**0,033**	94,7	0,328	80,6	0,213	68,0	0,217	67,4	0,194
	Quit	9	36,7		61,1		38,9		77,8		67,1		56,1		48,9	
	Less	17	47,4		82,4		51,5		90,2		75,5		57,4		58,2	
	More	5	82,0		92,5		70,0		100		72,0		70,0		66,0	
																
*Physical activity*	As much	18	73,9	**0,000**	82,6	0,963	81,9	**0,027**	88,9	0,531	74,9	0,795	66,9	0,462	68,1	0,344
	Quit	4	12,5		81,3		37,5		75,0		69,0		50,0		46,3	
	Less	32	57,5		85,5		53,1		93,8		77,8		62,0		60,2	
	More	2	80,0		81,3		75,0		100		76,0		70,0		55,0	
																
*Hobbies*	As much	35	62,3	0,888	85,4	0,663	72,9	**0,009**	93,3	0,751	77,3	0,667	63,6	0,838	59,3	0,166
	Quit	1	70,0		100		100		100		56,0		60,0		55,0	
	Less	19	56,3		80,3		39,5		86,0		75,4		61,3		68,2	
	More	1	60,0		100		75,0		100		72,0		80,0		20,0	
																
*Visiting*	As much	38	67,6	**0,000**	92,1	**0,000**	67,1	0,141	95,6	0,087	81,8	**0,001**	68,2	**0,003**	65,3	0,054
	Quit	3	8,3		62,5		25,0		66,7		65,3		33,3		33,3	
	Less	15	52,3		68,3		56,7		84,4		64,0		56,0		57,7	

FF: physical functioning, SF: social functioning, RLf: role limitations - physical, RLe: role limitations - emotional, MH: mental health, Vit: vitality, GH: general health.

^#^Based on the HADS (none: <8 points, possible: 8-10 points, probable: >10 points)

### Caregiver strain

Nearly half of the patients (49%) had a partner. In this study most partners experience no strain difference compared to before stroke. Only 4% of the caregivers experienced considerable strain. Of the partners experiencing some form of strain, 2 CSI factors were most problematic, namely, 'changes in family life' and 'the confining nature of caregiving'. Although most caregivers experienced no burden, most were often afraid to go away and leave their partner at home alone.

Gender, social situation, housekeeping, physical activities, hobbies, visiting, depression and anxiety variables were compared to caregiver strain. There were no significant relations between these variables and the amount of caregiver strain (Fisher's exact test).

Table 5 shows the relationship between age, functioning and the HRQOL of stroke patients and caregiver burden. There is a moderate and negative correlation for caregiver strain with the ADL dependency of the patient and with physical functioning from the health-related quality of life.

**Table 5:** Comparison of caregiver strain with age, functioning and HRQOL.

	CSI (R*)
* Age*	0,176
*Barthel Index*	-0,434
*Physical functioning (FF)*	-0,575
*Social functioning (SF)*	-0,241
*Role limitations - physical (Rlf)*	-0,296
*Role limitations - emotional (Rle)*	0,030
* Mental Health (MH)*	-0,200
*Vitality (Vit)*	-0,312
*Bodily Pain (BP)*	-0,028

*Spearman Correlation

## Discussion

The aim of this study was to provide insight into the functioning of patients who returned to their home immediately after discharge from hospital, one year post stroke. Insight into the functioning of these patients can ultimately be used to determine whether GPs can assert a positive influence on functioning and quality of life.

One year post stroke most patients are no longer ADL dependent. Although 31% of the patients had lower BI scores, the median score of all patients at discharge and after one year did not differ significantly. Older patients and those more ADL dependent at discharge had a worse ADL dependency after one year. This study also showed a relationship between ADL independence and the loss of social contacts. This is consistent with other research [32,33]. Whether better aftercare and rehabilitation can improve ADL independence and thus improve the number of social contacts should be investigated.

Anxiety and depression are not related to a decline in dependency, but for depression the p-value is only 0.057, so when we repeat this study in a larger group of patients, there possibly could be a significant relation to being more dependent.

45% of patients could perform the same housekeeping tasks as before stroke. A possible explanation is that a lot of women spouses take care of housekeeping, meaning that there is minimal change in the housekeeping tasks for men who suffered a stroke.

Before this study we expected that the prevalence of depression and anxiety disorder in stroke patients would be higher than normal. House et al. [34] confirmed this hypothesis. 2% of the elderly in the Netherlands currently have depression or have just had depression [27,28]. There was an indication for depression in this study in 13% of the patients. This is much higher than in the general elderly population. 11% of the elderly aged 65 to 75 years in the Netherlands have had an anxiety disorder in the past year [31]. In this study 9% of the patients had an indication for anxiety disorder. However, this is a point prevalence and not a year prevalence, meaning that full comparison is not possible.

Since depression and anxiety disorders are often present in stroke patients and because this presence is related to HRQOL, it is recommended that psychic functioning be surveyed more effectively and that these conditions be treated when possible. HRQOL can thus be influenced positively. The percentages in this study, however, can only be regarded as an indication. For definite diagnoses of depression and anxiety disorders, more extensive tests should be performed. Furthermore, the HADS questions are sometimes suggestive. It is possible that patients provide socially desirable answers out of shame on account of these suggestions. More research should be done to confirm that more attention should be paid to psychical functioning.

The health-related quality of life in this study group was high. Other studies have reported lower HRQOL after one year, although they included all patients with ischemic stroke rather than only primary care patients. They possibly studied more severely impaired stroke patients. In contrast, in this study the mean age is higher [35,36]. Taiwanese older patients one year post ischemic stroke were interviewed at home. These patients on average seemed to have lower HRQOL than the patients in our study [37].

The HRQOL of the study population was almost the same as that of the Dutch elderly population. Only the 'role limitations - emotional' and 'bodily pain' health scales scored differently. In the present study stroke patients seem to experience fewer 'role limitations - emotional' and less 'bodily pain'. This is possibly due to the impact of the stroke in the preceding year. Directly after their strokes, patients experienced more difficulties in functioning in multiple aspects of life. The improvement in functioning could lead to a more positive view of pain and emotional functioning.

As stated, patients with signs of anxiety disorder or depression have a significantly worse HRQOL for the 'social functioning', 'role limitations - emotional', 'mental health' and 'vitality' items. Other studies confirm that psychical functioning is an important factor in HRQOL [6,36].

In this study HRQOL and ADL independence were correlated. Lower ADL independence correlates with worse scores for 'physical functioning', 'role limitations - physical', 'mental health' and 'vitality' in the SF-36. Other studies of HRQOL also show a relationship between difficulties in physical functioning and lower HRQOL [35,37,38].

Patients who cannot do housekeeping, exercise, hobbies or visit family and friends also show significantly lower HRQOL on multiple aspects.

With this in mind, HRQOL could increase further were patients to receive support to resume activities and where psychic problems are recognized and treated. There are indications that brief psychosocial intervention and antidepressant treatment reduces post-stroke depression and improves functional outcomes [39,40]. Another possibility is to start community-based rehabilitation programmes. This could increase stroke patients' activity levels and provide greater satisfaction [41-43].

We expected the caregiver strain burden to be high. However, only 4% of spouses experienced considerable strain. Other studies show much higher percentages of caregiver strain. Visser-Meily et al. (2005) found considerable burden experienced by as many as 54% of spouses [44]. However, the study included younger patients and patients from rehabilitation centres with a lower Barthel Index and thus a lower level of ADL independence. A study by Bugge et al. (1999) shows that burden increases over time. After half a year, 37% of spouses experience caregiver strain [45]. Blake et al. (2003) found that 40% of caregivers experience burden [46]. These last two studies were performed with GP patients and compare better with our study population. Predictive factors for burden in these two studies were patient functioning, psychological factors related to the caregiver and social support. The relationship between functioning and caregiver strain can be confirmed in this study. Moreover, there is a negative correlation between some aspects of the SF-36 and caregiver strain. Burden seems to be very low in this study. However, in this study relatively few caregivers were interviewed, the patients were well functioning and the spouses and patients were interviewed together, which may have clouded its findings. Follow-up research should include more spouses, individual interviews and a control group to determine whether the strain is due to the stroke or due to other factors, such as age. However, in this study no relationship between age and burden was found.

The mortality in our study was relatively low. Seven percent of the patients died in the first year post stroke. Other studies report mortality around 30% [4,47]. Mortality in this study is also low when compared to the Bamford et al. study. However, they included all ischemic stroke patients and patients of all ages. The severity of the strokes in this study was relatively low, given the NIHSS scores at admission to hospital.

This study has several limitations. We studied all patients who went home immediately after discharge from hospital. However, many patients first go to a rehabilitation centre or nursing home to rehabilitate. These patients - when discharged from the rehabilitation centre - also come under GP care and probably have worse outcomes than the patients in this study. Therefore, the results in this study cannot be generalized for all stroke patients living at home.

The study group was relatively small because of the selection criteria (patients who went home immediately after discharge from hospital and patients from a single hospital). To make stronger generalizations, the study group should be larger and patients from multiple hospitals should be interviewed.

Furthermore, the present study group has a very high mean age and thus it is possible that decline in functioning is partially due to normal ageing. To determine the respective roles of stroke and normal ageing in relation to a decline in functioning, a control group should be used.

Finally, patients who refused to participate in this study were not considered. The patients who refused told us that they were doing fine and that a visit was not necessary. Another patient was in hospital for a hip fracture. Theoretically, these patients could have been those whose physical and psychical functioning was very bad, though the number of patients in the group was very small.

A strong point in this study is the method of data acquisition. We visited patients at home and completed the questionnaires together. A lot of information is gained through conversation, not only by talking but also by demonstration.

Another strength is the fact that one researcher visited all of the patients and the fact that we used standardized questionnaires. This avoids the risk of different interpretations of the results.

## Conclusions

To conclude, this study provides some insight into aspects of the wellbeing of the elderly one year post stroke. Most patients are ADL independent and HRQOL is about the same as in the general Dutch elderly population. Improvements can be envisioned in mental functioning and in maintaining habits and daily occupations. When physical functioning is poor at discharge from hospital, people function even worse after one year. All the former aspects seem to influence quality of life. Although burden is very low on average in this study, caregiver strain seems to be higher when the HRQOL of patients is lower.

Despite the low number of patients, our results indicate that paying more attention to rehabilitation (maintaining habits and daily occupations) and psychical functioning in the first year, could improve HRQOL after one year. Further research with a larger group of patients is needed to confirm whether influencing rehabilitation and psychical functioning actually improves quality of life in stroke patients. There may be a role for GPs in this, for example by regularly monitoring the physical and psychical problems of patients with a short questionnaire and by performing more interventions to improve mood and participation.

## Competing interests

The authors declare that they have no competing interests.

## Authors' contributions

LW, WR and KM initiated the study. LW wrote the protocol. WR and KM supervised data collection. LW, WR, KG and KM wrote the manuscript. LW and KG performed statistical analysis. All the authors have read and reviewed the final manuscript.

## Pre-publication history

The pre-publication history for this paper can be accessed here:

http://www.biomedcentral.com/1471-2377/11/42/prepub
